# Gene Expression Profile Analysis of T1 and T2 Breast Cancer Reveals Different Activation Pathways

**DOI:** 10.1155/2013/924971

**Published:** 2013-02-28

**Authors:** Margit L. H. Riis, Xi Zhao, Fateme Kaveh, Hilde S. Vollan, Anne-Jorunn Nesbakken, Hiroko K. Solvang, Torben Lüders, Ida R. K. Bukholm, Vessela N. Kristensen

**Affiliations:** ^1^Department of Surgery, Akershus University Hospital, 1478 Lørenskog, Norway; ^2^Institute for Clinical Medicine, Faculty of Medicine, University of Oslo, 0318 Oslo, Norway; ^3^Department of Radiology, School of Medicine, Stanford Center for Cancer Systems Biology, Stanford University, Stanford, CA 94305-5488, USA; ^4^Department of Genetics, Institute for Cancer Research, Oslo University Hospital Radiumhospitalet, 0310 Oslo, Norway; ^5^Department of Clinical Molecular Biology and Laboratory Sciences (EpiGen), Akershus University Hospital, 1478 Lørenskog, Norway; ^6^Department of Pathology, Akershus University Hospital, 1478 Lørenskog, Norway; ^7^Institute of Health Promotion, Akershus University Hospital, 1478 Lørenskog, Norway

## Abstract

Breast cancers today are of predominantly T1 (0.1 ≥ 2.0 cm) or T2 (>2 ≤ 5 cm) categories due to early diagnosis. Molecular profiling using microarrays has led to the notion of breast cancer as a heterogeneous disease both clinically and molecularly. Given the prognostic power and clinical use of tumor size, the purpose of this study was to search for molecular signatures characterizing clinical T1 and T2. In total 46 samples were included in the discovery dataset. After adjusting for hormone receptor status, lymph node status, grade, and tumor subclass 441 genes were differently expressed between T1 and T2 tumors. Focal adhesion and extracellular matrix receptor interaction were upregulated in the smaller tumors while p38MAPK signaling and immune-related pathways were more dominant in the larger tumors. The T-size signature was then tested on a validation set of 947 breast tumor samples. Using the T-size expression signatures instead of tumor size leads to a significant difference in risk for distant metastases (*P* < 0.001). If further confirmed, this molecular signature can be used to select patients with tumor category T1 who may need more aggressive treatment and patients with tumor category T2 who may have less benefit from it.

## 1. Introduction

Breast cancer is by far the most frequent cancer among women, and ranks second overall [1]. Guidelines for breast cancer treatment are based upon classical clinicopathological parameters: age, tumor size, grade, lymph node status, and histological type; in addition to hormone receptor status [2]. Lymph node (N) status is the most powerful single indicator of breast cancer prognosis [3], while tumor size, categorized into four groups (T1–4) is the second strongest indicator and is independent of lymph node status [3]. Here we attempted to identify the molecular background behind this prognostic effect of tumor size.

Mammographic screening has led to breast cancer diagnosis at preclinical stage and, as a consequence, most diagnosed cases present as T1 or T2, with significantly better survival in T1 tumors [4]. Nevertheless, T1 tumors may also give recurrence or metastases. Chemotherapy and hormonal treatment reduce the risk of recurrence or distant metastases by approximately 30% and according to the current guidelines whether a tumor is T1 or T2 is a critical factor in treatment decision. However, 70–80% of patients would have survived without adjuvant treatment [5]. How to distinguish the patients that would benefit from adjuvant treatment would therefore be of great value to the patient preventing possible severe side effects, and to the community saving from unnecessary health expenses.

Microarray technology has enabled to study thousands of genes simultaneously. Interpretation of the data requires advanced statistical analysis [6] and there has been a long way to clinical implication [7]. Hierarchical clustering has been the simplest algorithm applied to organize both genes and samples into groups based on similarity of gene expression [8]. Based on this, breast cancers have been separated into several molecular subclasses [9]. This implies breast cancer as a heterogeneous group of malignancy with distinct molecular signature. The molecular subgroups have been studied in respect of clinical implication and are significantly correlated to overall survival and recurrence of disease [10]. As opposed to this unsupervised approach, the principle of supervised analysis is one where predictive models are built based on existing knowledge of the clinical characteristics [11, 12]. This methodology has also been used to establish a good and a poor prognosis profile which is a more powerful predictor of outcome in young patients with breast cancer than the standard systems based on clinicohistological criteria [10, 13].

Since one of the most crucial factors of treatment decision is tumor size, we aimed to find a gene expression profile which will best characterize the two most common groups of tumors: T1 and T2. We first identified the most differentially expressed genes between T1 and T2 tumors in 46 patients and characterized the biological pathways active in each category. We then validated this gene list on other publically available datasets.

## 2. Materials and Methods

### 2.1. Sample Collection

Core needle biopsies were collected at Akershus University Hospital, Norway, between 2003 and 2007. The tumors were detected clinically or through screening by mammography. The samples were taken under ultrasound guidance and immediately placed into RNA later (Sigma Aldrich, St. Louis, MO, USA). The stabilized samples were stored at −80°C. The study is approved by the Regional Committee for Medical and Health Research Ethics (REK) and all women included have signed a consent form.

**Figure 1:**
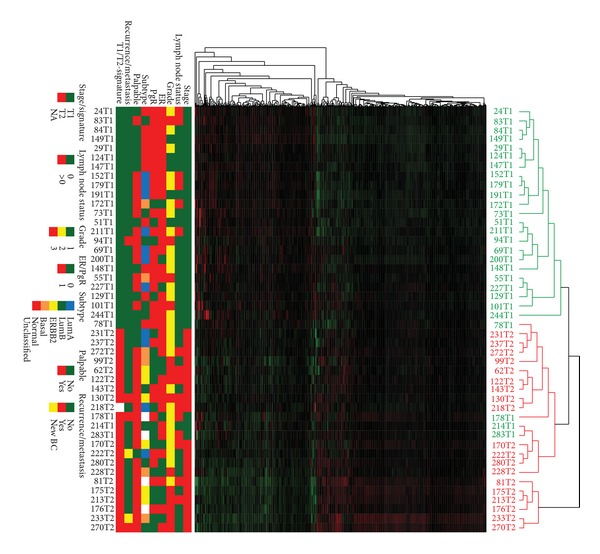
Unsupervised hierarchical clustering using the 441 significant probes after adjustment for clinical parameters. Genes are listed vertically and each patient is represented in the columns. Clinical T1 tumors are shown in green while clinical T2 tumors are shown in red.

This study includes in total 46 samples, 27 T1 and 19 T2 infiltrating ductal carcinomas. T1 lesion is defined as no more than 2 cm in size while T2 lesion is defined as above 2 cm up to 5 cm. The clinical parameters of the tumors are summarized in Table 1 and Figure 1. Within the T1 group two women had recurrence or metastasis, both of these are deceased. In addition two other women in this group are deceased but without cancer specific death. In the T2 group two patients had metastasis or recurrence, one of these has deceased. In addition there was one more case of mortality in this group; this patient developed malignant melanoma with liver metastasis which was the probable cause of death. Among the women in the T2 group two patients developed a new breast cancer.

### 2.2. RNA Isolation

Frozen biopsies were homogenized in 600 *μ*L Trizol (Invitrogen, Carlsbad, CA, USA) using a 5 mm steal bead (Qiagen, Hilden, Germany) and a Mixer Mill MM301 (Retsch, Haan, Germany) at 20 Hz for 2 min before adding an additional 600 *μ*L Trizol, followed by 240 *μ*L chloroform (Sigma Aldrich). After centrifugation (15 min, 12000 ×g, 4°C) the upper aqueous phase was transferred to a new tube and RNA precipitated by adding an equal volume of isopropanol. After centrifugation the pellet was washed 2-3 times with 75% ethanol and dissolved in 40 *μ*L RNase-free water (Ambion, Austin, TX, USA). Concentration was measured using NanoDrop (Thermo Fisher Scientific, Waltham, MA, USA) and RNA quality assayed on a 2100 Bioanalyzer (Agilent, Santa Clara, CA, USA). The purified RNA was stored at –80°C.

### 2.3. Microarray Analysis

10 *μ*g total RNA was amplified using Amino Allyl MessageAmp II aRNA Amplification Kit (Ambion) followed by posttranscriptional labeling with CyDye Cy3 or Cy5 (GE HealthCare, Chalfont St. Giles, UK). As a reference probe universal human reference RNA (UHR; Stratagene, La Jolla, CA, USA) was amplified and labeled as above. Amplification and labeling efficiency were controlled on the NanoDrop. Labeled cRNA corresponding to 20 picomoles cyanine dye each of experimental and reference samples were mixed and hybridized to Agilent Whole Human Genome Oligo Microarrays (1 × 44 k format) per manufacturer's protocol (Ver. 4.1). After hybridization at 60°C for 17 hours the arrays were washed and scanned using an Agilent scanner.

Data collection and quality assessment were performed using Agilent Feature Extraction software v8.5 with default parameters. Preprocessing was performed using JExpress Pro v2.7 [14]. Poor spots flagged by Feature Extraction were filtered out and Loewess normalization applied. Missing values were calculated with the LSimpute function for genes with less than 5% missing values. To find significant changes of genes/probes between the two tumor stages, Statistical Analysis of Microarray (SAM) [15] was applied. To adjust for lymph node status, differential grade, estrogen and progesterone receptor status, and breast cancer subtype, a partial least squares regression analysis was performed with the pls package in R [16, 17]. To find biological functions pathway analysis was performed for the up- and downregulated genes in T1 and T2 using DAVID [18, 19]. The upregulated genes in T1 are simultaneously the downregulated genes in T2 and vice versa. To confirm and visualize the differential expression between T1 and T2 tumors, unsupervised hierarchical clustering using the genes/probes significantly deregulated by SAM was performed in JExpress Pro. The microarray data have been submitted to the ArrayExpress Archive (http://www.ebi.ac.uk/microarray-as/ae/), accession number: E-MTAB-1049.

### 2.4. Validation Set

To validate the T-size signature in an independent dataset, we collected expression profiles of 947 breast tumor samples [20] from six published microarray datasets [21–26] with updated followups. The datasets are accessible from NCBI's Gene Expression Omnibus (GEO, http://www.ncbi.nlm.nih.gov/geo/) with the following identifiers; GSE6532 for the Loi dataset [21], GSE3494 for the Miller dataset [22], GSE1456 for the Pawitan dataset [23], GSE7390 for the Desmedt dataset [24], and GSE2603 for the Minn dataset [25]. The Chin dataset [26] is available from ArrayExpress (http://www.ebi.ac.uk/) with identifier E-TABM-158.

These datasets were all measured on Human Genome HG-U133A Affymetrix arrays. Each dataset was RMA-normalized [27] and median centered per gene. All overlapping samples from the Desmedt and Loi datasets were excluded. The datasets were then merged based on the common probes. Gene centering has been shown to effectively remove many data set specific biases allowing effective integration of multiple data sets [28]. The merged dataset did not show batch effect after pulling (see Zhao et al. [20]).

### 2.5. Gene Signatures Evaluated on the Validation Set

For the T-size signature, tumors in the validation set were assigned to either T1-like group or T2-like group using the nearest of the T-size expression centroids (distances computed using correlation to the centroids). The risk group assignment corresponded to the label of the centroid with the highest correlation. We did not apply a correlation cutoff when assigning risk groups; every sample received a classification based on the T-size signature.

We further compared the prognostic power of the T-size signature with eight established prognostic gene signatures for breast cancer. These are Intrinsic [9, 29, 30], PAM50 [31], 70-gene or MammaPrint (Agendia, Amsterdam, The Netherlands) [13, 32], 76-gene [33], Genomic-Grade-Index (GGI) [21, 34], 21-gene-Recurrence-Score (RS) or Oncotype DX (Genomic Health Inc., Redwood City, CA) [35], Wound-Response (WR) signature [36, 37], and Hypoxia signature [38, 39]. All included gene signatures were implemented using the original algorithms. For Intrinsic and PAM50, in addition to subtype classification, a risk score per sample was computed by linear combination of the centroid correlations in ROR-S model (Risk-Of-Relapse scores by Subtype alone) [31]. A pseudo Oncotype DX Recurrence Score per patient was computed by the unscaled Recurrence Score [35]. For 76-genes, GGI and RS, rather than assigning risk groups based on published cutoffs, we used a population-based approach in which a fixed proportion of the population was assigned to each risk group. The proportions were derived from previous datasets associated with individual signatures [24, 34, 35]. We found this necessary as our analyses differed from the original methods in technical or methodological manners (see details in Zhao et al. [20]). To make a fair comparison across signatures, we assessed the signatures on the full dataset.

### 2.6. Survival Analysis

The signatures were evaluated for prediction of Distant Metastasis Free Survival (DMFS). A total of 912 patients on the validation set (*n* = 947) had available DMFS status with median followup for 81 months. The Kaplan-Meier survival curves were plotted for the corresponding risk groups. The differences in survival probabilities associated with the risk groups were tested by a logrank test.

A *likelihood ratio test* was used to assess the significance of the overall effect in a univariate comparison of predictors. *Deviance* was used to check the goodness of the model fit. The marginal contribution by a single predictor in the univariate setting was evaluated using the *proportion of variation explained* in the outcome variable (PVE) [40], which is an indicator for the importance of covariates in the Cox model. The* Hazard Ratio* (HR) was used as an accuracy measure for the risk group prediction for different predictors. The *concordance index* (C-index) [41] was computed to assess the predictive discrimination ability of each of the predictors in the corresponding univariate Cox model. For a multivariate comparison of predictors, the relative importance of a covariate in a multivariate Cox model was measured by the partial PVE.

## 3. Results

After preprocessing 36,669 genes were included for further analyses. Comparing the gene expression profiles between T1 and T2 tumors and using partial least squares regression (PLS) analysis to adjust for lymph node status, differential grade, hormone receptor status, and breast cancer subtype, yielded 441 genes differentially expressed genes at FDR <1% (Supplementary Table  S1 available online at http://dx.doi.org/10.1155/2013/924971). Unsupervised hierarchical clustering using these 441 probes resulted in T1 and T2 tumors to cluster for most part separately (Figure 1) except four T1 tumors that clustered with the T2 tumors. One of these patients developed metastasis to the lung and to the bone, and later died. Another one is still alive but had bilateral breast cancer in addition to primary lung cancer. The last two patients in this group are free of recurrence and metastasis.

### 3.1. Pathway Analysis

**Figure 2:**
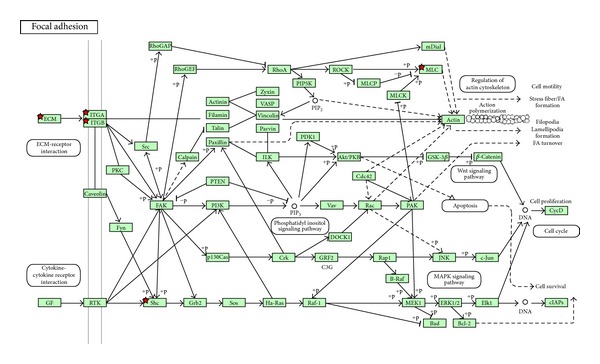
Focal adhesion is one of the pathways upregulated in clinical T1 and downregulated in clinical T2. Red star implies the gene is upregulated in clinical T1 and downregulated in clinical T2.

**Figure 3:**
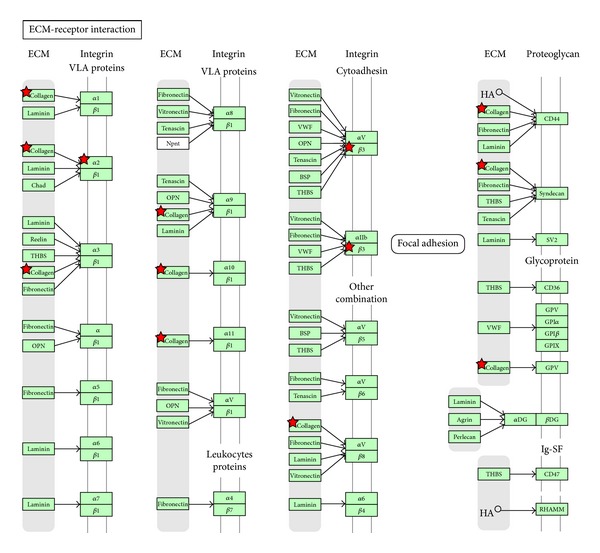
ECM receptor interactions are upregulated in clinical T1 and downregulated in clinical T2. Red star implies the gene is upregulated in clinical T1 and downregulated in clinical T2.

To further study the differences in genes between T1 and T2 tumors, we performed pathway analysis. Of the 441 significant probes, 184 probes were upregulated in T1 (downregulated in T2), and 257 probes were downregulated in T1 (upregulated T2). The genes upregulated in T1 were enriched for several pathways (Table 2), including Focal Adhesion (Figure 2) and ECM- (extracellular matrix) receptor interaction (Figure 3). Among the important upregulated genes are several collagens and integrins, and p27 (cyclin dependent kinase inhibitor 1B).

**Figure 4:**
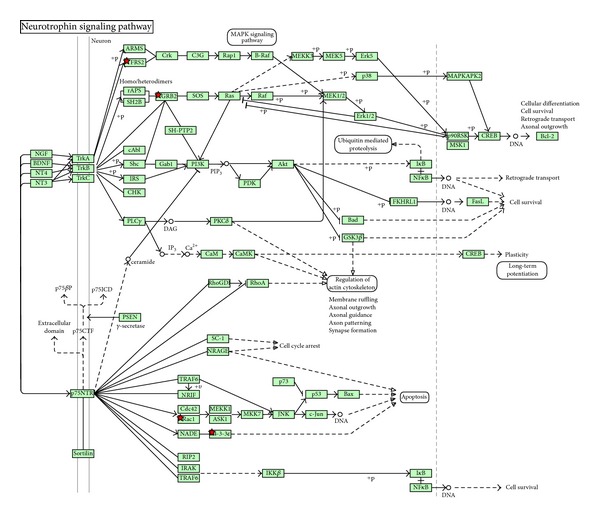
Neurotrophin signaling pathway is among the pathways downregulated in clinical T1 and upregulated in clinical T2. Red stars imply genes that are downregulated in clinical T1 and upregulated in T2.

The downregulated genes in T1, upregulated in T2 were enriched for important pathways like Neurotrophin signaling pathway (Figure 4), p38MAPK signaling pathway, and several pathways involved in immune response (Table 3). Important genes in these pathways are MNK1 (MAP kinase interacting serine/threonine-protein kinase 1), GRB2 (Growth factor receptor bound protein 2), RAC1 (ras-related C3 botulinum toxin substrate 1), and several immune-related genes, such as IFN, IL6, MHCII, and complement component 1.

### 3.2. Validation of the T-Size Signature

**Figure 5:**
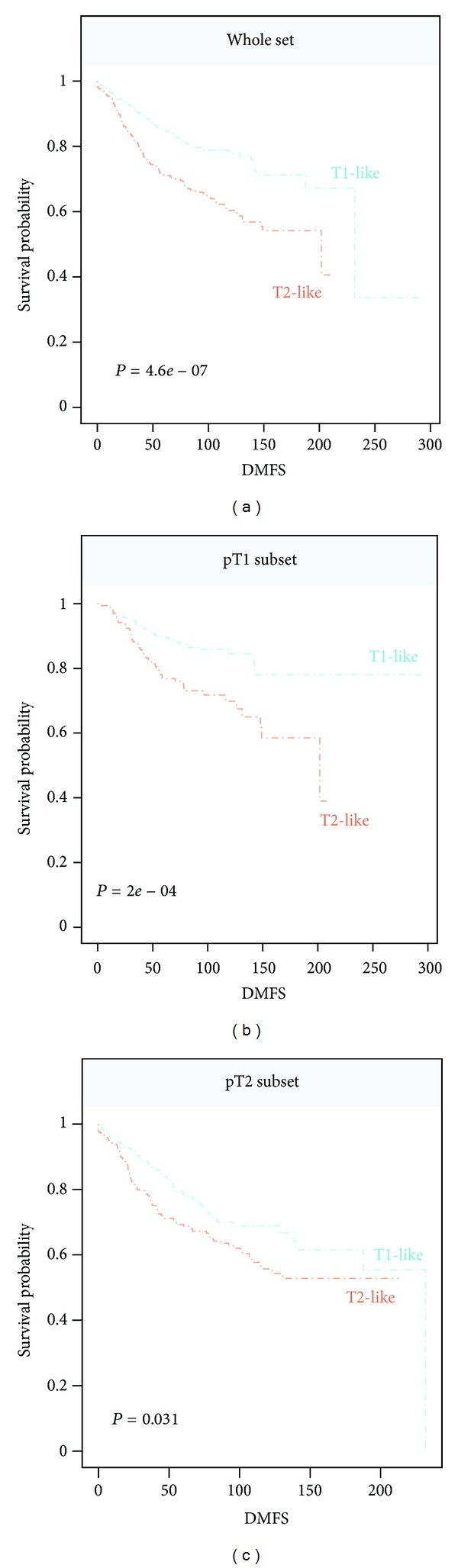
T-size signature for prediction for Distant Metastasis Free Survival (DMFS) on the Affy947 breast cancer dataset. Survival probabilities associated with the risk groups are shown by Kaplan Meier plot. A total of 912 patients had available DMFS status. Follow-up time is shown in month. (a) T-size signature in the complete set. (b) In pT1 tumor subset. (c) In pT2 tumor subset.

In the validation of the T-size signature, a total of 480 samples were called as T1-like, and 467 were classified as T2-like. For all signatures except Hypoxia on the complete set for DMFS (*n* = 912), differences in DMFS between risk groups were highly significant (not shown; see Zhao et al. [20]). Specifically for the T-size signature, the separation between T1-like group and T2-like group was highly significant (*P* < 0.001; Figure 5(a)) with T2-like group associated with higher risk for distant metastasis. We also observed highly significant separation of two risk groups for DMFS in the patient group with pT1 size tumors (*n* = 440; *P* < 0.001; Figure 5(b)); while in the pT2 tumor subgroup (*n* = 459), T-size signature achieved less significant separation for the risk prediction (*P* = 0.031; Figure 5(c)).

We performed univariate analysis for the T-size signature and clinical parameters including tumor size (1–3), node status (positive versus negative), ER status (positive versus negative), and histological grade (1–3), respectively. The performance comparisons by using the likelihood ratio test, the deviance, the *proportion of variation explained *(PVE), the *concordance index* (C-index), and the *Hazard Ratio* (HR) are summarized in Table 4. A multivariate Cox model was used to simultaneously assess the T-size signature and the included clinical parameters in the study. Due to the known association between ER status and survival, we included ER status as stratification variable (Table 5).

## 4. Discussion

Approximately 15% of all women diagnosed with breast cancer die from their disease within 5 years of diagnosis [42] despite having been treated according to national clinical guidelines [2]. Both genomic and clinical variables should be induced in a common algorithm to yield the most accurate prediction model. Microarray has made it possible to study thousands of genes simultaneously. This generates information about gene expression profiles that can be computed in different ways. One of these is the clustering of patients according to the gene expression in their tumors. The majority of the gene lists are generated to distinguish patients from being subject to unnecessary adjuvant treatment or with the intention of individualizing therapy and treatment.

Several genetic signatures have been presented [13, 23, 32–34]. This work has led to the development of special kits such as MammaPrint (Agendia, Amsterdam, The Netherlands) [13, 32] and Oncotype DX (Genomic Health Inc., Redwood City, CA, USA) [35]. By combining information from multiple gene signatures, one would potentially increase the prediction power and bring out an overall picture of this disease. Zhao et al. aimed to develop an analytical framework that allows us to utilize the combined strength from individual gene signatures [43]. Such a framework and the resulting model will be broadly applicable for survival prediction across heterogeneous tumor groups capturing a broad spectrum of biological aspects. The tumor size associated signature presented here has the purpose to identify the molecular characteristics associated to size and does not claim to provide prognostic index superior to the existing ones. The signature specific difference in DMFS within the T1 subgroup and the T2 subgroup, shown here, are used only to suggest that it can be used as supplementary information to tumor size.

Most first generation signatures are good for predicting prognosis in early stage breast cancer. There is only a minor overlap in genes in the different signatures [44], but they produce similar risk group assignment in the same dataset. Proliferation and the level of proliferation-related genes are the strongest prognostic factors in ER positive cancer. Proliferation-related genes are often highly expressed in ER negative cancers, so in the first generation signatures almost all ER negative cancers seem to have poor prognosis. It was initially meant that these prognostic signatures could replace the classical histopathological findings, but meta-analysis has revealed that tumor size and lymph node status give prognostic information independent of the molecular signatures [45]. 

The present study attempts to identify, independent of grade, receptor status and lymph node status, the molecular signature, and the underlying biological pathways associated to tumor size, which is an objective property without possibility of interobserver disagreement. The most significant pathways upregulated in T1 compared to T2 are focal adhesion, ECM-receptor interaction, and two organ specific pathways (Table 2). Important genes occur at several steps in these pathways. One of these genes being P27(Kip) (cyclin dependent kinase inhibitor 1B). The cell-cycle regulating protein p27^Kip1^ (p27) has dual roles by acting as both a cdk inhibitor and as an assembly factor for different cdk complexes. Loss of p27 has been linked to malignant features in different tumors [46]. High levels of p27 are expressed in normal human mammary epithelium, but loss of p27 is frequent in breast cancer and has been demonstrated to have prognostic implications [47]. Patients with tumors expressing low levels of p27 were associated with poor prognosis, and it is especially pronounced in hormone-receptor positive tumors [48]. HER2 positive primary breast cancers often reveal low levels of p27 [49]. As mentioned, in our material p27 is upregulated in T1 tumors compared to T2 tumors and this is in coherence with earlier studies. Thus this could be a possible marker, among others, that could be used to select the T1 tumors that have a greater possibility of recurrence. The lower p27, the worse prognosis, consequently requiring stronger treatment.

Pathways downregulated in T1 and upregulated in T2 are shown in Table 3. These are all pathways associated with the immune response, and a majority of the actual downregulated genes are immune response related genes, like IFN, IL6, MHC II, and Complement component 1. This is consistent with a more aggressive lesion that requires more effort from the immune system. Among the genes downregulated in T1 tumors compared to T2 tumors is GRB2. Grb2 is an adaptor protein that is essential for a variety of cellular functions and acts as a critical downstream intermediary in several oncogenic signaling pathways [50]. In human breast cancer cells Grb2 is overexpressed. In an unpublished work we have demonstrated that there is a significant difference in the expression of this gene in normal tissue and breast cancer tissue, and also in normal tissue adjacent to tumor. The role of Grb2 as a signal transducer for several oncogenic growth factor receptors and the broad involvement of Grb2 in multiple steps of the metastasis cascade make it a good target for antitumor therapeutic strategies [50]. Like for p27, maybe this gene could be measured in the patients with smaller tumors to select those with worse prognosis.

RAC1 (ras-related C3 botulinum toxin substrate 1) may represent an attractive target. Rac GTPases, small G-proteins widely implicated in tumorigenesis and metastasis, transduce signals from tyrosine-kinase, G-protein-coupled receptors (GPCRs), and integrins, and control a number of essential cellular functions including motility, adhesion, and proliferation. In breast cancer cells Rac1 is a downstream effector of ErbB receptors and mediates migratory responses by ErbB1/EGFR ligands such as EGF or TGF*α* and ErbB3 ligands such as heregulins [51]. This gene is a potential target for use in therapy of breast cancer.

## 5. Conclusions

In summary we show here that there is a molecular profile that is associated to tumor size. Thus a gene-expression signature-based approach combined with the classical TNM classification as well as analysis of key genes may pave the way to improved individualized therapy.

## Supplementary Material

Supplementary Table S1 shows the differently expressed genes (FDR <2% by SAM) between T1 and T2 breast cancer tumors after adjustment for lymph node status, hormone receptor status, differential grade, and breast cancer subtype.

## Figures and Tables

**Table 1:** Summary of patient characteristics.

Sample	Stage	Size/cm	Grade	Node	ER	PgR	Recurrence/metastasis	Deceased
24	T1	0.8	2	1	Positive	Positive		
29	T1	0.8	2	0	Positive	Positive		
51	T1	1.0	1	0	Positive	Negative		
55	T1	0.8	3	0	Positive	Positive		
62	T2	2.5	3	2	Negative	Negative		
69	T1	1.4	2	0	Positive	Positive		
73	T1	1.8	2	0	Positive	Positive		
78	T1	1.2	2	0	Positive	Positive		
81	T2	2.3	2	1	Positive	Positive		
83	T1	1.9	3	1	Positive	Positive		
84	T1	1.0	2	0	Positive	Positive		
94	T1	1.9	3	0	Positive	Negative	Yes	Yes
99	T2	4.0	3	0	Negative	Negative		
101	T1	1.9	3	0	Positive	Positive		Yes
122	T2	3.4	3	1	Positive	Negative		Yes
124	T1	0.9	1	0	Positive	Positive		
129	T1	0.8	2	0	Positive	Negative		
130	T2	2.2	3	2	Positive	Positive	Yes	Yes
143	T2	2.8	2	0	Positive	Positive		
147	T1	1.0	1	0	Positive	Positive		
148	T1	0.9	2	0	Positive	Positive		
149	T1	1.0	1	0	Positive	Positive		
152	T1	1.5	2	1	Positive	Positive		
170	T2	4.0	2	1	Negative	Negative		
172	T1	1.9	2	1	Negative	Negative		Yes
175	T2	2.5	3	0	Negative	Negative		
176	T2	2.7	3	0	Positive	Positive		
178	T1	1.2	2	1	Negative	Positive	Yes	Yes
179	T1	1.8	2	1	Positive	Positive		
191	T1	1.4	1	0	Positive	Positive		
200	T1	1.5	2	0	Negative	Positive		
211	T1	1.7	2	2	Positive	Positive		
213	T2	3.0	3	1	Negative	Negative		
214	T1	1.2	2	0	Positive	Negative		
218	T2	2.1	1-2	1	Positive	Negative		
222	T2	2.1	2	0	Positive	Positive	New BC	
227	T1	1.8	2	0	Negative	Positive		
228	T2	3.0	3	0	Negative	Negative		
231	T2	2.1	2	0	Positive	Negative		
233	T2	3.0	3	0	Negative	Negative	New BC	
237	T2	2.1	2	0	Positive	Positive		
244	T1	0.6	2	0	Positive	Positive		
270	T2	3.0	3	0	Positive	Negative	Yes	
272	T2	2.3	2	1	Positive	Negative		
280	T2	3.2	2	0	Negative	Positive		
283	T1	1.3	2	1	Positive	Negative		

**Table 2:** Upregulated pathways in T1 breast cancer tumors compared to T2 tumors.

Term	Genes	Count	%	*P* value	Benjamini
Focal adhesion (KEGG_PATHWAY)	MYL7, ITGA2, ITGB3, COL4A6, SHC4	5	3.5	3.6*E* − 2	8.6*E* − 1
Arrhythmogenic right ventricular cardiomyopathy (ARVC) (KEGG_PATHWAY)	LEF1, ITGA2, ITGB3	3	2.1	8.2*E* − 2	9.0*E* − 1
Small cell lung cancer (KEGG_PATHWAY)	ITGA2, ITGB3, COL4A6	3	2.1	9.7*E* − 2	8.4*E* − 1
ECM-receptor interaction (KEGG_PATHWAY)	CDKN1B, ITGA2, COL4A6	3	2.1	9.7*E* − 2	8.4*E* − 1

**Table 3:** Downregulated pathways in T1 breast cancer tumors compared to T2 tumors.

Term	Genes	Count	%	*P* value	Benjamini
Neurotrophin signaling pathway (KEGG_PATHWAY)	YWHAZ, GRB2, RAC1, YWHAQ, FRS2	5	2.8	4.2*E* − 2	9.8*E* − 1
p38 MAPK Signaling Pathway (BIOCARTA)	GRB2, RAC1, MKNK1	3	1.7	5.0*E* − 2	9.8*E* − 1
Prion diseases (KEGG_PATHWAY)	C1QA, C1QB, C1QC	3	1.7	5.3*E* − 2	9.1*E* − 1
Jak-STAT signaling pathway (KEGG_PATHWAY)	OSM, IFNA2, GRB2, IL10RA, IL4R	5	2.8	8.1*E* − 2	9.2*E* − 1
Systemic lupus erythematosus (KEGG_PATHWAY)	C1QA, C1QB, HLA-DPB1, C1QC	4	2.2	8.7*E* − 2	8.7*E* − 1
Toll-like receptor signaling pathway (KEGG_PATHWAY)	IFNA2, MYD88, TICAM1, RAC1	4	2.2	9.1*E* − 2	8.2*E* − 1

**Table 4:** Univariate comparison of predictors.

Covariate	HR [95% CI]	*P*	PVE	Deviance	*C*
T-size signature					
(Overall effect)		4.30*E* − 07	2.76*E* − 02	25.55	0.58
T2-like (versus T1-like)	1.92 [1.48–2.48]	7.22*E* − 07			
Tumor size					
(Overall effect)		5.63*E* − 08	3.62*E* − 02	33.38	0.60
2 (versus 1)	1.95 [1.50–2.55]	7.83*E* − 07			
3 (versus 1)	3.39 [1.96–5.88]	1.37*E* − 05			
Node					
(Overall effect)		2.40*E* − 06	2.46*E* − 02	22.24	0.58
+ (versus −)	1.89 [1.46–2.45]	1.35*E* − 06			
ER					
(Overall effect)		2.07*E* − 02	5.85*E* − 03	5.35	0.54
+ (versus −)	0.72 [0.55–0.94]	1.78*E* − 02			
Histological grade					
(Overall effect)		2.45*E* − 04	2.11*E* − 02	16.63	0.60
2 (versus 1)	1.78 [1.15–2.77]	1.04*E* − 02			
3 (versus 1)	2.37 [1.52–3.69]	1.78*E* − 02			

**Table 5:** Multivariate comparison of predictors.

Covariate	HR [95% CI]	*P*	Partial PVE
T-size signature			
T2-like (versus T1-like)	1.70 [1.25–2.32]	8.03*E* − 04	1.42*E* − 02
Tumor size			
2 (versus 1)	1.74 [1.29–2.35]	2.72*E* − 04	1.62*E* − 02
3 (versus 1)	2.07 [0.97–4.40]	5.98*E* − 02	1.62*E* − 02
Node			
+ (versus −)	1.68 [1.24–2.28]	8.11*E* − 04	1.25*E* − 02
Histological grade			
2 (versus 1)	1.45 [0.92–2.29]	1.14*E* − 01	2.05*E* − 02
3 (versus 1)	1.49 [0.91–2.47]	1.16*E* − 01	2.05*E* − 02
