# Supplementary material for: Gene Expression Profile Analysis of T1 and T2 Breast Cancer Reveals Different Activation Pathways
Source: ISRN Oncol. 2013 Feb 28;2013:924971. doi: 10.1155/2013/924971 (PMC3603375; doi:10.1155/2013/924971)
Supplement: Supplementary file 1 — Supplementary Table S1 shows the differently expressed genes (FDR <2% by SAM) between T1 and T2 breast cancer tumors after adjustment for lymph node status, hormone receptor status, differential grade, and breast cancer subtype. [file 924971.f1.doc]

**Supplementary table S1** Differently expressed genes between T1 and T2 breast cancer tumors after adjustment for lymoh node status, hormone receptor status, differential grade, and breast cancer subtype. The expression values are given as log_2_(expr[sample]/expr[reference]).

| **ProbeName** | **GeneName** | **Avg expr T1** | **Avg expr T2** | **Fold Change** | **q-value (%)** | **T1 vs T2** |
| --- | --- | --- | --- | --- | --- | --- |
| A_23_P157795 | CTNNAL1 | -2,15 | -2,65 | 1,42 | 0,00 | Up |
| A_23_P161352 | PTPLA | -1,18 | -1,65 | 1,38 | 0,00 | Up |
| A_23_P167256 | PHF17 | -0,99 | -1,43 | 1,36 | 0,00 | Up |
| A_23_P211273 | TMPRSS3 | 0,34 | -0,29 | 1,55 | 0,00 | Up |
| A_23_P318300 | ZAK | 0,13 | -0,66 | 1,72 | 0,00 | Up |
| A_23_P35617 | PLCE1 | 0,22 | -0,64 | 1,82 | 0,00 | Up |
| A_23_P404211 | ENST00000267430 | -0,25 | -0,58 | 1,25 | 0,00 | Up |
| A_23_P62932 | ATP1B1 | 0,68 | -0,52 | 2,30 | 0,00 | Up |
| A_24_P241276 | EXOSC8 | -1,17 | -1,60 | 1,35 | 0,00 | Up |
| A_24_P398691 | C13orf6 | -0,04 | -0,30 | 1,20 | 0,00 | Up |
| A_24_P56317 | MBNL2 | 0,51 | -0,17 | 1,61 | 0,00 | Up |
| A_24_P699737 | AK022339 | -0,99 | -1,38 | 1,31 | 0,00 | Up |
| A_24_P81841 | CDKN1B | -0,21 | -1,20 | 1,99 | 0,00 | Up |
| A_24_P89426 | APOM | -1,63 | -2,16 | 1,44 | 0,00 | Up |
| A_32_P228699 | PRDM10 | -0,17 | -0,45 | 1,22 | 0,00 | Up |
| A_32_P4792 | AK057820 | -0,48 | -0,96 | 1,40 | 0,00 | Up |
| A_23_P100711 | PMP22 | 1,53 | 0,76 | 1,71 | 0,20 | Up |
| A_23_P111303 | RBM16 | -0,32 | -0,68 | 1,28 | 0,20 | Up |
| A_23_P148969 | LRRC40 | -1,01 | -1,43 | 1,34 | 0,20 | Up |
| A_23_P14975 | C16orf48 | -0,05 | -0,38 | 1,26 | 0,20 | Up |
| A_23_P152678 | EPPB9 | -0,12 | -0,65 | 1,44 | 0,20 | Up |
| A_23_P19987 | IMP-3 | -2,63 | -3,13 | 1,40 | 0,20 | Up |
| A_23_P354798 | FLJ32452 | -0,20 | -0,66 | 1,37 | 0,20 | Up |
| A_23_P3602 | THC2303868 | -0,31 | -0,81 | 1,41 | 0,20 | Up |
| A_23_P394874 | DSCR10 | 0,03 | -0,20 | 1,17 | 0,20 | Up |
| A_23_P433369 | C14orf125 | 0,06 | -0,33 | 1,32 | 0,20 | Up |
| A_23_P59738 | MYL7 | -0,05 | -0,26 | 1,15 | 0,20 | Up |
| A_23_P72680 | CDV1 | -0,65 | -1,04 | 1,31 | 0,20 | Up |
| A_23_P99710 | KIAA0586 | -0,58 | -0,91 | 1,25 | 0,20 | Up |
| A_24_P100673 | LOC51234 | 0,08 | -0,38 | 1,38 | 0,20 | Up |
| A_24_P128001 | ZNF395 | -0,16 | -0,87 | 1,64 | 0,20 | Up |
| A_24_P849467 | ZNF585A | 0,18 | -0,06 | 1,18 | 0,20 | Up |
| A_24_P913561 | C12orf2 | -0,48 | -0,79 | 1,24 | 0,20 | Up |
| A_24_P920319 | LOC58486 | -0,07 | -0,61 | 1,45 | 0,20 | Up |
| A_32_P156024 | USP54 | -0,19 | -0,69 | 1,41 | 0,20 | Up |
| A_32_P234145 | RaLP | -0,56 | -0,89 | 1,26 | 0,20 | Up |
| A_32_P76060 | HSPC129 | -0,54 | -0,85 | 1,24 | 0,20 | Up |
| A_23_P217379 | COL4A6 | -0,54 | -1,22 | 1,60 | 0,23 | Up |
| A_23_P30315 | TRIM7 | -0,84 | -1,41 | 1,49 | 0,23 | Up |
| A_23_P47034 | HHEX | -0,90 | -1,31 | 1,33 | 0,23 | Up |
| A_23_P55616 | ENST00000321925 | -0,19 | -0,54 | 1,27 | 0,23 | Up |
| A_23_P77310 | ZFP106 | 0,17 | -0,17 | 1,26 | 0,23 | Up |
| A_24_P108301 | RTTN | -0,42 | -0,71 | 1,23 | 0,23 | Up |
| A_24_P116233 | C13orf1 | -0,34 | -0,70 | 1,28 | 0,23 | Up |
| A_24_P178475 | A_24_P178475 | -1,30 | -1,66 | 1,28 | 0,23 | Up |
| A_24_P281665 | LOC554226 | 0,69 | -0,02 | 1,64 | 0,23 | Up |
| A_24_P576445 | TBC1D8 | -0,10 | -0,64 | 1,45 | 0,23 | Up |
| A_24_P902100 | AL359559 | -0,40 | -0,80 | 1,32 | 0,23 | Up |
| A_24_P920447 | C14orf132 | 0,38 | -0,14 | 1,44 | 0,23 | Up |
| A_24_P922838 | AI570381 | 0,12 | -0,03 | 1,11 | 0,23 | Up |
| A_24_P928522 | DST | 0,47 | -0,04 | 1,42 | 0,23 | Up |
| A_24_P938403 | JPH3 | 0,02 | -0,18 | 1,15 | 0,23 | Up |
| A_24_P942822 | MDH1B | 0,38 | 0,16 | 1,17 | 0,23 | Up |
| A_32_P137399 | ENST00000335142 | -0,34 | -0,58 | 1,19 | 0,23 | Up |
| A_32_P211141 | LOC90624 | -1,05 | -1,43 | 1,30 | 0,23 | Up |
| A_32_P93894 | A_32_P93894 | 0,55 | 0,11 | 1,36 | 0,23 | Up |
| A_23_P392435 | KBTBD6 | -0,25 | -0,53 | 1,21 | 0,42 | Up |
| A_23_P39955 | ACTG2 | 0,95 | -0,01 | 1,95 | 0,42 | Up |
| A_23_P85521 | ZNF265 | -0,40 | -0,73 | 1,26 | 0,42 | Up |
| A_24_P356406 | PAFAH1B1 | 0,29 | -0,07 | 1,28 | 0,42 | Up |
| A_24_P401645 | AP4S1 | -0,19 | -0,43 | 1,18 | 0,42 | Up |
| A_24_P889473 | AF085920 | -0,05 | -0,23 | 1,13 | 0,42 | Up |
| A_24_P913168 | AA835868 | -0,02 | -0,19 | 1,13 | 0,42 | Up |
| A_32_P108420 | A_32_P108420 | 0,22 | 0,02 | 1,15 | 0,42 | Up |
| A_32_P115375 | THC2308298 | -0,18 | -0,47 | 1,22 | 0,42 | Up |
| A_32_P129912 | LOC441383 | -0,01 | -0,36 | 1,28 | 0,42 | Up |
| A_32_P154121 | THC2340670 | 0,24 | 0,02 | 1,17 | 0,42 | Up |
| A_32_P27706 | THC2435127 | -0,14 | -0,60 | 1,38 | 0,42 | Up |
| A_32_P330691 | AF390550 | 0,24 | -0,01 | 1,19 | 0,42 | Up |
| A_23_P110031 | ZNF589 | 0,02 | -0,26 | 1,21 | 0,46 | Up |
| A_23_P136504 | SLC25A21 | -0,48 | -0,66 | 1,14 | 0,46 | Up |
| A_23_P161628 | MUS81 | -0,02 | -0,39 | 1,29 | 0,46 | Up |
| A_23_P164638 | ZNF419 | 0,16 | -0,13 | 1,22 | 0,46 | Up |
| A_23_P212339 | FYCO1 | 1,00 | 0,56 | 1,35 | 0,46 | Up |
| A_23_P213045 | LEF1 | -0,82 | -1,17 | 1,28 | 0,46 | Up |
| A_23_P300479 | AY239294 | -0,15 | -0,39 | 1,18 | 0,46 | Up |
| A_23_P313828 | PRR6 | -1,54 | -2,05 | 1,42 | 0,46 | Up |
| A_23_P57268 | CXADR | -0,69 | -1,54 | 1,79 | 0,46 | Up |
| A_23_P65217 | TGDS | -0,96 | -1,20 | 1,18 | 0,46 | Up |
| A_24_P13715 | FBXO22 | -0,05 | -0,27 | 1,17 | 0,46 | Up |
| A_24_P209765 | DCTN3 | 0,09 | -0,26 | 1,27 | 0,46 | Up |
| A_24_P296765 | ZNF211 | 0,28 | -0,01 | 1,22 | 0,46 | Up |
| A_24_P306585 | CD359021 | -0,59 | -0,88 | 1,22 | 0,46 | Up |
| A_24_P317135 | EXOSC7 | -0,48 | -0,77 | 1,23 | 0,46 | Up |
| A_24_P371053 | ORMDL1 | 0,10 | -0,32 | 1,34 | 0,46 | Up |
| A_24_P461881 | BF978194 | 0,10 | -0,08 | 1,14 | 0,46 | Up |
| A_32_P174121 | T35134 | -0,18 | -0,60 | 1,34 | 0,46 | Up |
| A_32_P18448 | BM806490 | 0,05 | -0,22 | 1,21 | 0,46 | Up |
| A_32_P29632 | CXADR | -0,82 | -1,75 | 1,92 | 0,46 | Up |
| A_32_P44775 | C9orf85 | -0,60 | -0,85 | 1,19 | 0,46 | Up |
| A_23_P117286 | CLN5 | 0,64 | 0,28 | 1,28 | 0,62 | Up |
| A_23_P203972 | FZD10 | -0,60 | -1,06 | 1,37 | 0,62 | Up |
| A_23_P364837 | VPS13D | 0,44 | 0,17 | 1,21 | 0,62 | Up |
| A_23_P3856 | ZFP1 | -0,61 | -0,92 | 1,24 | 0,62 | Up |
| A_23_P65031 | DNCL1 | 0,26 | 0,68 | 0,75 | 0,62 | Down |
| A_24_P176714 | EPPB9 | -0,18 | -0,53 | 1,27 | 0,62 | Up |
| A_24_P268993 | LEAP-2 | -0,62 | -0,94 | 1,25 | 0,62 | Up |
| A_24_P289709 | HBG1 | -0,37 | -0,69 | 1,25 | 0,62 | Up |
| A_24_P483956 | A_24_P483956 | 0,13 | 0,82 | 0,62 | 0,62 | Down |
| A_24_P76018 | AK097724 | 0,14 | -0,03 | 1,12 | 0,62 | Up |
| A_24_P80915 | THC2428671 | -0,39 | -0,65 | 1,19 | 0,62 | Up |
| A_24_P936947 | A_24_P936947 | -0,40 | -0,66 | 1,20 | 0,62 | Up |
| A_24_P941759 | KIAA1333 | -0,32 | -0,57 | 1,19 | 0,62 | Up |
| A_32_P172755 | THC2409451 | -0,06 | -0,32 | 1,19 | 0,62 | Up |
| A_32_P191285 | THC2319894 | -0,06 | -0,25 | 1,14 | 0,62 | Up |
| A_32_P46495 | BX090412 | 0,39 | 0,04 | 1,28 | 0,62 | Up |
| A_23_P150325 | AD031 | 0,13 | -0,21 | 1,26 | 0,72 | Up |
| A_23_P160200 | ZNF643 | -0,10 | -0,44 | 1,27 | 0,72 | Up |
| A_23_P27107 | TM4SF5 | -1,06 | -1,43 | 1,29 | 0,72 | Up |
| A_23_P2732 | KIAA1704 | -0,60 | -0,84 | 1,18 | 0,72 | Up |
| A_23_P351734 | NPHP4 | -0,22 | -0,49 | 1,21 | 0,72 | Up |
| A_23_P36567 | DTX1 | 0,36 | 0,03 | 1,26 | 0,72 | Up |
| A_23_P372874 | S100A13 | 0,66 | 0,16 | 1,41 | 0,72 | Up |
| A_23_P424603 | DKFZp547D2210 | 0,01 | 0,17 | 0,89 | 0,72 | Down |
| A_23_P67432 | ZNF583 | 0,08 | -0,19 | 1,20 | 0,72 | Up |
| A_24_P140475 | ARGBP2 | 0,89 | 0,38 | 1,43 | 0,72 | Up |
| A_24_P201936 | GTF2A1 | -0,41 | -0,72 | 1,24 | 0,72 | Up |
| A_24_P202857 | C20orf38 | 0,08 | 0,28 | 0,87 | 0,72 | Down |
| A_24_P215240 | C10orf63 | 0,16 | -0,04 | 1,15 | 0,72 | Up |
| A_24_P247978 | ZNF589 | 0,15 | -0,07 | 1,17 | 0,72 | Up |
| A_24_P374943 | CXADR | -0,56 | -1,01 | 1,36 | 0,72 | Up |
| A_24_P649507 | THC2438003 | -0,19 | -0,34 | 1,11 | 0,72 | Up |
| A_24_P655833 | BC047708 | -0,35 | -0,60 | 1,18 | 0,72 | Up |
| A_24_P659099 | THC2341659 | 0,28 | 0,07 | 1,15 | 0,72 | Up |
| A_24_P810735 | AX721128 | -0,02 | -0,36 | 1,26 | 0,72 | Up |
| A_24_P880143 | THC2317029 | 0,13 | 0,33 | 0,87 | 0,72 | Down |
| A_24_P912300 | A_24_P912300 | -0,08 | -0,27 | 1,14 | 0,72 | Up |
| A_24_P929003 | ITGB3 | 0,09 | -0,07 | 1,12 | 0,72 | Up |
| A_32_P101779 | SEC14L3 | 0,04 | -0,06 | 1,08 | 0,72 | Up |
| A_32_P125135 | LOC440224 | -0,75 | -1,29 | 1,46 | 0,72 | Up |
| A_32_P129540 | LOC440110 | -0,76 | -1,12 | 1,29 | 0,72 | Up |
| A_32_P229746 | DNAJB6 | -0,18 | 0,30 | 0,72 | 0,72 | Down |
| A_32_P447001 | FLJ27352 | 0,37 | -0,05 | 1,34 | 0,72 | Up |
| A_32_P453321 | MGC33556 | -0,87 | -1,15 | 1,21 | 0,72 | Up |
| A_23_P103897 | SPATA1 | 0,27 | 0,49 | 0,86 | 0,92 | Down |
| A_23_P108143 | GAMT | -0,35 | -0,89 | 1,45 | 0,92 | Up |
| A_23_P130482 | ZNF211 | 0,45 | 0,18 | 1,20 | 0,92 | Up |
| A_23_P13524 | DKFZp586C1924 | -0,49 | -0,78 | 1,22 | 0,92 | Up |
| A_23_P135742 | C1orf170 | 0,17 | -0,01 | 1,13 | 0,92 | Up |
| A_23_P138376 | RAB18 | 0,26 | 0,70 | 0,73 | 0,92 | Down |
| A_23_P138760 | CLCF1 | 0,05 | -0,23 | 1,21 | 0,92 | Up |
| A_23_P15765 | TNRC6C | 0,12 | 0,27 | 0,90 | 0,92 | Down |
| A_23_P157963 | C9orf39 | -0,21 | -0,38 | 1,12 | 0,92 | Up |
| A_23_P161563 | RAB38 | -0,52 | -0,94 | 1,34 | 0,92 | Up |
| A_23_P164284 | CLDN7 | 1,56 | 2,16 | 0,66 | 0,92 | Down |
| A_23_P168351 | HEY2 | 0,08 | -0,23 | 1,24 | 0,92 | Up |
| A_23_P17456 | SIRPB1 | 0,01 | 0,18 | 0,89 | 0,92 | Down |
| A_23_P18282 | DLEC1 | 0,20 | 0,02 | 1,13 | 0,92 | Up |
| A_23_P200126 | MKNK1 | 0,49 | 0,77 | 0,83 | 0,92 | Down |
| A_23_P201086 | ARF1 | -0,16 | 0,31 | 0,72 | 0,92 | Down |
| A_23_P20122 | ZC3HAV1 | -0,02 | 0,22 | 0,84 | 0,92 | Down |
| A_23_P202100 | A_23_P202100 | 0,21 | 0,47 | 0,83 | 0,92 | Down |
| A_23_P215406 | RAC1 | -0,18 | 0,40 | 0,67 | 0,92 | Down |
| A_23_P252371 | RBBP8 | -0,33 | -1,08 | 1,68 | 0,92 | Up |
| A_23_P27147 | ANAPC11 | -0,09 | 0,47 | 0,68 | 0,92 | Down |
| A_23_P27515 | PLD3 | 0,27 | 0,65 | 0,77 | 0,92 | Down |
| A_23_P304511 | ZNF397 | 0,40 | 0,11 | 1,22 | 0,92 | Up |
| A_23_P308552 | DLX3 | 0,13 | 0,56 | 0,74 | 0,92 | Down |
| A_23_P324718 | SYNJ1 | -0,36 | -0,68 | 1,25 | 0,92 | Up |
| A_23_P336198 | GLCCI1 | -0,36 | -0,83 | 1,38 | 0,92 | Up |
| A_23_P341532 | SETDB2 | 0,27 | 0,03 | 1,18 | 0,92 | Up |
| A_23_P371495 | ARG99 | -0,22 | -0,91 | 1,61 | 0,92 | Up |
| A_23_P376096 | TICAM1 | 0,11 | 0,38 | 0,83 | 0,92 | Down |
| A_23_P40192 | CDH22 | 0,68 | 1,09 | 0,75 | 0,92 | Down |
| A_23_P409502 | NALP9 | 0,15 | 0,38 | 0,85 | 0,92 | Down |
| A_23_P413193 | C10orf78 | -0,42 | -0,73 | 1,24 | 0,92 | Up |
| A_23_P424472 | CDC14A | -0,07 | -0,72 | 1,58 | 0,92 | Up |
| A_23_P47282 | ST14 | 0,77 | 1,21 | 0,74 | 0,92 | Down |
| A_23_P501134 | LIG3 | 0,12 | 0,46 | 0,79 | 0,92 | Down |
| A_23_P77833 | PGS1 | -0,02 | 0,18 | 0,87 | 0,92 | Down |
| A_23_P97064 | FBXO6 | 0,21 | 0,45 | 0,85 | 0,92 | Down |
| A_23_P98477 | CCDC15 | -0,09 | -0,29 | 1,14 | 0,92 | Up |
| A_24_P15182 | ENST00000273011 | -0,08 | 0,26 | 0,79 | 0,92 | Down |
| A_24_P166443 | HLA-DPB1 | 3,18 | 3,81 | 0,65 | 0,92 | Down |
| A_24_P239811 | UBXD5 | 0,58 | 0,23 | 1,28 | 0,92 | Up |
| A_24_P268786 | MYNN | -0,09 | -0,37 | 1,21 | 0,92 | Up |
| A_24_P269101 | NEUROG1 | 0,34 | 0,65 | 0,81 | 0,92 | Down |
| A_24_P271323 | SLC2A10 | 0,50 | 0,94 | 0,74 | 0,92 | Down |
| A_24_P323815 | MYCBP2 | 0,15 | -0,22 | 1,29 | 0,92 | Up |
| A_24_P34568 | FLJ38377 | 0,12 | 0,41 | 0,82 | 0,92 | Down |
| A_24_P380567 | CALN1 | 0,10 | 0,29 | 0,88 | 0,92 | Down |
| A_24_P383199 | ENST00000260303 | 0,10 | 0,30 | 0,87 | 0,92 | Down |
| A_24_P453544 | AY239294 | -0,23 | -0,50 | 1,21 | 0,92 | Up |
| A_24_P714316 | A_24_P714316 | 0,43 | 0,92 | 0,71 | 0,92 | Down |
| A_24_P75158 | PTAR1 | -0,33 | -0,71 | 1,30 | 0,92 | Up |
| A_24_P7584 | LY6G5C | 0,23 | 0,51 | 0,83 | 0,92 | Down |
| A_24_P780393 | LOC442002 | 0,86 | 1,31 | 0,73 | 0,92 | Down |
| A_24_P85881 | GPR113 | 0,27 | 0,59 | 0,81 | 0,92 | Down |
| A_24_P930469 | A_24_P930469 | 0,21 | 0,38 | 0,89 | 0,92 | Down |
| A_24_P933613 | THC2304870 | -0,17 | -0,38 | 1,15 | 0,92 | Up |
| A_32_P115555 | AA742526 | 0,57 | 0,96 | 0,77 | 0,92 | Down |
| A_32_P118586 | FLJ34969 | 0,32 | 0,03 | 1,22 | 0,92 | Up |
| A_32_P124125 | AA451906 | 0,04 | 0,61 | 0,68 | 0,92 | Down |
| A_32_P161033 | LOC440776 | -0,53 | -0,88 | 1,27 | 0,92 | Up |
| A_32_P163472 | A_32_P163472 | 0,20 | -0,01 | 1,16 | 0,92 | Up |
| A_32_P173177 | A_32_P173177 | 0,24 | -0,06 | 1,23 | 0,92 | Up |
| A_32_P178800 | THC2402993 | -0,59 | -1,23 | 1,56 | 0,92 | Up |
| A_32_P18547 | C21orf57 | -0,06 | -0,42 | 1,29 | 0,92 | Up |
| A_32_P2148 | ENST00000339726 | -0,37 | -0,68 | 1,24 | 0,92 | Up |
| A_32_P228268 | ENST00000280576 | 0,08 | 0,26 | 0,89 | 0,92 | Down |
| A_32_P2452 | ARG99 | 0,17 | -0,39 | 1,47 | 0,92 | Up |
| A_32_P42236 | LOC440234 | 0,20 | -0,43 | 1,55 | 0,92 | Up |
| A_32_P461386 | AK057355 | 0,05 | 0,23 | 0,88 | 0,92 | Down |
| A_32_P731227 | A_32_P731227 | 0,14 | 0,33 | 0,88 | 0,92 | Down |
| A_32_P9518 | BX090181 | 0,40 | 0,65 | 0,84 | 0,92 | Down |
| A_23_P100156 | FLJ20507 | 0,25 | 0,45 | 0,87 | 1,21 | Down |
| A_23_P102060 | SSFA2 | -0,33 | -1,10 | 1,71 | 1,21 | Up |
| A_23_P109964 | NYD-SP17 | 0,09 | 0,25 | 0,89 | 1,21 | Down |
| A_23_P114707 | H6PD | 0,35 | 0,60 | 0,84 | 1,21 | Down |
| A_23_P115118 | BMP8A | 1,01 | 1,54 | 0,69 | 1,21 | Down |
| A_23_P115124 | TRIM11 | 0,20 | 0,65 | 0,74 | 1,21 | Down |
| A_23_P115202 | CRNN | 0,28 | 0,52 | 0,85 | 1,21 | Down |
| A_23_P118370 | AK022252 | 0,15 | 0,37 | 0,86 | 1,21 | Down |
| A_23_P119266 | DNASE2 | 0,24 | 0,52 | 0,83 | 1,21 | Down |
| A_23_P123060 | ITGB8 | 0,20 | 0,42 | 0,86 | 1,21 | Down |
| A_23_P125844 | A_23_P125844 | 0,09 | 0,23 | 0,90 | 1,21 | Down |
| A_23_P125977 | C1QG | 1,16 | 1,71 | 0,68 | 1,21 | Down |
| A_23_P126917 | IGSF3 | 0,13 | 0,30 | 0,89 | 1,21 | Down |
| A_23_P128855 | SLC39A2 | 0,09 | 0,27 | 0,88 | 1,21 | Down |
| A_23_P129556 | IL4R | 0,44 | 0,86 | 0,75 | 1,21 | Down |
| A_23_P131723 | YWHAQ | -0,57 | -0,13 | 0,73 | 1,21 | Down |
| A_23_P133904 | MCCD1 | 0,02 | 0,27 | 0,84 | 1,21 | Down |
| A_23_P135219 | GPR21 | 0,34 | 0,59 | 0,84 | 1,21 | Down |
| A_23_P135465 | FLJ32675 | 0,32 | 0,57 | 0,84 | 1,21 | Down |
| A_23_P137366 | C1QB | 2,77 | 3,54 | 0,59 | 1,21 | Down |
| A_23_P142506 | GADD45B | 0,36 | 0,76 | 0,76 | 1,21 | Down |
| A_23_P14295 | C14orf162 | 0,16 | 0,38 | 0,86 | 1,21 | Down |
| A_23_P15357 | LGALS3BP | 0,39 | 0,80 | 0,75 | 1,21 | Down |
| A_23_P15394 | CD68 | 0,43 | 0,95 | 0,70 | 1,21 | Down |
| A_23_P156505 | IRF4 | 0,24 | 0,53 | 0,82 | 1,21 | Down |
| A_23_P158969 | SLC39A11 | 0,03 | 0,38 | 0,78 | 1,21 | Down |
| A_23_P159721 | GPR50 | 0,25 | 0,46 | 0,86 | 1,21 | Down |
| A_23_P16047 | GPR4 | 0,14 | 0,31 | 0,89 | 1,21 | Down |
| A_23_P161719 | CWF19L2 | -0,15 | -0,33 | 1,13 | 1,21 | Up |
| A_23_P161983 | MRGPRX4 | 0,17 | 0,37 | 0,87 | 1,21 | Down |
| A_23_P162199 | PIP5K2C | 0,33 | 0,61 | 0,82 | 1,21 | Down |
| A_23_P163200 | RIN3 | 0,10 | 0,25 | 0,91 | 1,21 | Down |
| A_23_P165521 | STRN | 0,15 | 0,36 | 0,86 | 1,21 | Down |
| A_23_P166408 | OSM | -0,01 | 0,25 | 0,83 | 1,21 | Down |
| A_23_P169257 | TNFSF8 | 0,25 | 0,46 | 0,86 | 1,21 | Down |
| A_23_P202156 | NFKB2 | 0,07 | 0,35 | 0,82 | 1,21 | Down |
| A_23_P204225 | A_23_P204225 | -0,08 | -0,21 | 1,10 | 1,21 | Up |
| A_23_P214944 | ALDH8A1 | 0,21 | 0,42 | 0,86 | 1,21 | Down |
| A_23_P215227 | DNAJB6 | -0,25 | 0,13 | 0,77 | 1,21 | Down |
| A_23_P216766 | HBLD2 | 0,21 | 0,44 | 0,85 | 1,21 | Down |
| A_23_P218977 | AFF4 | 0,19 | 0,38 | 0,87 | 1,21 | Down |
| A_23_P23037 | GJA8 | 0,13 | 0,31 | 0,88 | 1,21 | Down |
| A_23_P24135 | TACR2 | 0,22 | 0,44 | 0,85 | 1,21 | Down |
| A_23_P250251 | IFNA2 | 0,10 | 0,30 | 0,87 | 1,21 | Down |
| A_23_P256754 | GABRR1 | 0,20 | 0,39 | 0,88 | 1,21 | Down |
| A_23_P258703 | FOXP4 | -0,02 | 0,12 | 0,90 | 1,21 | Down |
| A_23_P2645 | SDS | 0,41 | 0,83 | 0,75 | 1,21 | Down |
| A_23_P300826 | C6orf136 | -0,05 | 0,23 | 0,83 | 1,21 | Down |
| A_23_P317244 | SPACA3 | 0,20 | 0,49 | 0,82 | 1,21 | Down |
| A_23_P321496 | C21orf99 | 0,47 | -0,03 | 1,41 | 1,21 | Up |
| A_23_P322395 | ZSCAN1 | 0,16 | 0,34 | 0,88 | 1,21 | Down |
| A_23_P329772 | VPS4A | 0,06 | 0,39 | 0,80 | 1,21 | Down |
| A_23_P332260 | PBOV1 | 0,18 | 0,38 | 0,88 | 1,21 | Down |
| A_23_P332937 | C19orf23 | 0,09 | 0,31 | 0,86 | 1,21 | Down |
| A_23_P348063 | SYNGR1 | 0,41 | 0,66 | 0,84 | 1,21 | Down |
| A_23_P352389 | FLJ25414 | 0,22 | 0,63 | 0,75 | 1,21 | Down |
| A_23_P3584 | LYPLA3 | 0,18 | 0,38 | 0,87 | 1,21 | Down |
| A_23_P35906 | CASP4 | 0,66 | 1,08 | 0,75 | 1,21 | Down |
| A_23_P362659 | MYD88 | 0,51 | 0,77 | 0,84 | 1,21 | Down |
| A_23_P369641 | LOC286073 | 0,27 | 0,51 | 0,85 | 1,21 | Down |
| A_23_P37535 | RAB8B | 0,25 | 0,45 | 0,87 | 1,21 | Down |
| A_23_P379630 | MGC15523 | 0,34 | 0,61 | 0,83 | 1,21 | Down |
| A_23_P384663 | MPP7 | 0,20 | 0,43 | 0,85 | 1,21 | Down |
| A_23_P387537 | PRRG3 | 0,24 | 0,46 | 0,86 | 1,21 | Down |
| A_23_P390443 | LOC284296 | 0,17 | 0,38 | 0,86 | 1,21 | Down |
| A_23_P398947 | SYT6 | -0,34 | -0,50 | 1,12 | 1,21 | Up |
| A_23_P399265 | STMN2 | 0,35 | 0,76 | 0,75 | 1,21 | Down |
| A_23_P40290 | A_23_P40290 | 0,27 | 0,44 | 0,89 | 1,21 | Down |
| A_23_P408195 | FLJ30834 | 0,25 | 0,51 | 0,83 | 1,21 | Down |
| A_23_P40919 | GPR128 | 0,17 | 0,37 | 0,87 | 1,21 | Down |
| A_23_P430785 | FRS2 | 0,11 | 0,32 | 0,87 | 1,21 | Down |
| A_23_P435477 | TMPRSS13 | 0,39 | 0,59 | 0,87 | 1,21 | Down |
| A_23_P44849 | KCTD13 | 0,05 | 0,22 | 0,89 | 1,21 | Down |
| A_23_P45999 | FBXO2 | 0,01 | -0,39 | 1,32 | 1,21 | Up |
| A_23_P47199 | SLC22A11 | 0,30 | 0,55 | 0,84 | 1,21 | Down |
| A_23_P49579 | NEUROD2 | 0,15 | 0,37 | 0,86 | 1,21 | Down |
| A_23_P58298 | LOC92345 | 0,09 | 0,26 | 0,89 | 1,21 | Down |
| A_23_P59452 | ABP1 | 0,12 | 0,31 | 0,87 | 1,21 | Down |
| A_23_P63343 | UTS2 | -2,16 | -2,64 | 1,40 | 1,21 | Up |
| A_23_P66497 | CACNG5 | 0,19 | 0,38 | 0,88 | 1,21 | Down |
| A_23_P68072 | WDR54 | -1,25 | -1,66 | 1,33 | 1,21 | Up |
| A_23_P69068 | PLXNA1 | 0,06 | 0,27 | 0,86 | 1,21 | Down |
| A_23_P75786 | SLC15A3 | 0,77 | 1,18 | 0,75 | 1,21 | Down |
| A_23_P77847 | GRB2 | 0,25 | 0,56 | 0,81 | 1,21 | Down |
| A_23_P78734 | MYH14 | 0,81 | 1,17 | 0,78 | 1,21 | Down |
| A_23_P80700 | KLHL18 | 0,12 | 0,32 | 0,87 | 1,21 | Down |
| A_23_P83659 | XPNPEP2 | 0,13 | 0,38 | 0,84 | 1,21 | Down |
| A_23_P86751 | ADARB2 | 0,53 | 0,97 | 0,74 | 1,21 | Down |
| A_23_P88466 | C15orf2 | 0,21 | 0,45 | 0,85 | 1,21 | Down |
| A_23_P88971 | CLN3 | 0,55 | 0,99 | 0,73 | 1,21 | Down |
| A_23_P89073 | ZNF23 | -0,07 | -0,42 | 1,28 | 1,21 | Up |
| A_23_P89931 | ZNF574 | 0,24 | 0,47 | 0,85 | 1,21 | Down |
| A_23_P96399 | AB027122 | 0,16 | 0,34 | 0,89 | 1,21 | Down |
| A_24_P107303 | IL10RA | 0,38 | 0,63 | 0,84 | 1,21 | Down |
| A_24_P118326 | THC2423311 | 0,32 | 0,79 | 0,73 | 1,21 | Down |
| A_24_P132787 | RAB18 | 0,48 | 0,84 | 0,78 | 1,21 | Down |
| A_24_P135628 | AK096715 | 0,60 | 0,91 | 0,81 | 1,21 | Down |
| A_24_P144082 | ENST00000319043 | 0,25 | 0,47 | 0,86 | 1,21 | Down |
| A_24_P15834 | TNXB | 0,02 | 0,35 | 0,80 | 1,21 | Down |
| A_24_P164854 | KIAA1271 | 0,33 | 0,53 | 0,87 | 1,21 | Down |
| A_24_P174613 | FBXW7 | 0,12 | -0,19 | 1,24 | 1,21 | Up |
| A_24_P186936 | ELN | 0,00 | 0,28 | 0,82 | 1,21 | Down |
| A_24_P204269 | MLL3 | 0,43 | 0,79 | 0,78 | 1,21 | Down |
| A_24_P206702 | ENST00000333405 | 0,20 | 0,44 | 0,85 | 1,21 | Down |
| A_24_P220472 | SPATA12 | 0,30 | 0,47 | 0,88 | 1,21 | Down |
| A_24_P222655 | C1QA | 1,74 | 2,39 | 0,64 | 1,21 | Down |
| A_24_P239578 | SLA | 0,13 | 0,37 | 0,85 | 1,21 | Down |
| A_24_P243741 | ADAM22 | 0,17 | 0,44 | 0,83 | 1,21 | Down |
| A_24_P246626 | ENST00000308384 | 3,26 | 3,89 | 0,65 | 1,21 | Down |
| A_24_P255654 | A_24_P255654 | -1,07 | -0,59 | 0,72 | 1,21 | Down |
| A_24_P256253 | AF242771 | 0,11 | 0,30 | 0,88 | 1,21 | Down |
| A_24_P260325 | LRAT | 0,11 | 0,30 | 0,88 | 1,21 | Down |
| A_24_P270769 | VPS35 | -0,30 | 0,22 | 0,69 | 1,21 | Down |
| A_24_P273708 | LOC391773 | 0,12 | 0,28 | 0,90 | 1,21 | Down |
| A_24_P281403 | OR4C46 | 0,26 | 0,49 | 0,85 | 1,21 | Down |
| A_24_P283834 | DRD2 | -0,01 | 0,26 | 0,83 | 1,21 | Down |
| A_24_P296424 | CLN3 | 0,51 | 0,95 | 0,74 | 1,21 | Down |
| A_24_P297526 | FLJ25143 | 0,14 | 0,31 | 0,89 | 1,21 | Down |
| A_24_P298723 | TTTY20 | 0,21 | 0,38 | 0,89 | 1,21 | Down |
| A_24_P301837 | HRH3 | 0,24 | 0,46 | 0,86 | 1,21 | Down |
| A_24_P305933 | TMCC3 | 0,27 | 0,58 | 0,81 | 1,21 | Down |
| A_24_P307042 | ENST00000327652 | 0,24 | 0,50 | 0,83 | 1,21 | Down |
| A_24_P307599 | AF210649 | 0,25 | 0,45 | 0,87 | 1,21 | Down |
| A_24_P308144 | AF119870 | 0,30 | 0,52 | 0,86 | 1,21 | Down |
| A_24_P312790 | SCN3B | 0,21 | 0,39 | 0,88 | 1,21 | Down |
| A_24_P33461 | LOC441120 | 0,31 | 0,56 | 0,84 | 1,21 | Down |
| A_24_P34611 | SIX3 | 0,00 | 0,19 | 0,87 | 1,21 | Down |
| A_24_P347880 | ALOXE3 | 0,22 | 0,46 | 0,85 | 1,21 | Down |
| A_24_P350959 | AF130108 | 0,28 | 0,56 | 0,83 | 1,21 | Down |
| A_24_P351420 | ZDHHC3 | 0,25 | -0,04 | 1,22 | 1,21 | Up |
| A_24_P359205 | PIGQ | -0,02 | 0,17 | 0,87 | 1,21 | Down |
| A_24_P370201 | EBI3 | 0,05 | 0,21 | 0,90 | 1,21 | Down |
| A_24_P378806 | NTSR1 | 0,22 | 0,47 | 0,84 | 1,21 | Down |
| A_24_P381945 | HMOX2 | 0,25 | 0,45 | 0,87 | 1,21 | Down |
| A_24_P389218 | ARHGEF5 | 0,27 | 0,52 | 0,84 | 1,21 | Down |
| A_24_P400507 | AER61 | -0,04 | -0,29 | 1,19 | 1,21 | Up |
| A_24_P403799 | NRIP2 | 0,34 | 0,60 | 0,83 | 1,21 | Down |
| A_24_P406301 | NDUFB2 | -0,08 | 0,35 | 0,74 | 1,21 | Down |
| A_24_P409182 | ENST00000303979 | 0,08 | 0,39 | 0,81 | 1,21 | Down |
| A_24_P410463 | TRPM6 | 0,20 | 0,39 | 0,88 | 1,21 | Down |
| A_24_P42768 | AF116667 | 0,37 | 0,57 | 0,87 | 1,21 | Down |
| A_24_P44341 | UGCGL2 | -0,53 | -0,73 | 1,15 | 1,21 | Up |
| A_24_P455610 | CR596789 | 0,17 | 0,41 | 0,85 | 1,21 | Down |
| A_24_P463779 | THC2330929 | 0,11 | 0,23 | 0,92 | 1,21 | Down |
| A_24_P479364 | THC2278340 | 0,04 | 0,20 | 0,89 | 1,21 | Down |
| A_24_P50601 | ENST00000355548 | 0,86 | 1,20 | 0,79 | 1,21 | Down |
| A_24_P522267 | AI674670 | 0,11 | 0,25 | 0,91 | 1,21 | Down |
| A_24_P528938 | AK022424 | 0,25 | 0,45 | 0,87 | 1,21 | Down |
| A_24_P537149 | A_24_P537149 | 0,22 | 0,53 | 0,81 | 1,21 | Down |
| A_24_P541213 | BX393065 | 0,28 | 0,45 | 0,89 | 1,21 | Down |
| A_24_P552450 | THC2315277 | 0,19 | 0,42 | 0,85 | 1,21 | Down |
| A_24_P587443 | THC2285086 | 0,27 | 0,48 | 0,87 | 1,21 | Down |
| A_24_P588897 | THC2277195 | 0,17 | -0,41 | 1,49 | 1,21 | Up |
| A_24_P58910 | C9orf33 | 0,06 | -0,05 | 1,08 | 1,21 | Up |
| A_24_P6381 | C5orf12 | 0,18 | 0,43 | 0,84 | 1,21 | Down |
| A_24_P659036 | MGC33692 | 0,14 | 0,39 | 0,84 | 1,21 | Down |
| A_24_P669220 | THC2314754 | 0,08 | 0,24 | 0,89 | 1,21 | Down |
| A_24_P678104 | STMN3 | -0,42 | -0,69 | 1,21 | 1,21 | Up |
| A_24_P68649 | RNPEP | -0,08 | 0,29 | 0,78 | 1,21 | Down |
| A_24_P72646 | FLJ14107 | 0,12 | 0,29 | 0,89 | 1,21 | Down |
| A_24_P741450 | THC2274876 | 0,23 | 0,44 | 0,86 | 1,21 | Down |
| A_24_P75460 | ENST00000312504 | -0,03 | -0,17 | 1,10 | 1,21 | Up |
| A_24_P770335 | THC2281896 | 0,30 | 0,60 | 0,82 | 1,21 | Down |
| A_24_P789471 | AA432215 | 0,22 | 0,44 | 0,85 | 1,21 | Down |
| A_24_P7965 | ESRRG | 0,09 | 0,34 | 0,85 | 1,21 | Down |
| A_24_P83787 | DISC1 | 0,23 | 0,47 | 0,84 | 1,21 | Down |
| A_24_P853124 | BG951912 | 0,07 | 0,27 | 0,87 | 1,21 | Down |
| A_24_P85606 | AF119898 | 0,11 | 0,25 | 0,91 | 1,21 | Down |
| A_24_P87746 | KIR2DS4 | 0,15 | 0,33 | 0,88 | 1,21 | Down |
| A_24_P884506 | AW028759 | -0,01 | 0,13 | 0,91 | 1,21 | Down |
| A_24_P891472 | A_24_P891472 | 0,19 | 0,40 | 0,87 | 1,21 | Down |
| A_24_P914573 | ENST00000314752 | 0,06 | 0,20 | 0,91 | 1,21 | Down |
| A_24_P918739 | ABHD1 | 0,26 | 0,50 | 0,84 | 1,21 | Down |
| A_24_P920712 | A_24_P920712 | 0,17 | 0,32 | 0,90 | 1,21 | Down |
| A_24_P920715 | A_24_P920715 | 0,06 | 0,20 | 0,91 | 1,21 | Down |
| A_24_P920795 | BI771744 | 0,02 | 0,17 | 0,90 | 1,21 | Down |
| A_24_P922624 | A_24_P922624 | 0,03 | 0,17 | 0,91 | 1,21 | Down |
| A_24_P923832 | ADAMTS13 | 0,10 | 0,40 | 0,81 | 1,21 | Down |
| A_24_P924543 | CB854194 | 0,22 | 0,41 | 0,88 | 1,21 | Down |
| A_24_P924739 | A_24_P924739 | 0,21 | 0,45 | 0,85 | 1,21 | Down |
| A_24_P924895 | BF826743 | 0,33 | 0,60 | 0,83 | 1,21 | Down |
| A_24_P925342 | MAN1C1 | 0,43 | 0,07 | 1,28 | 1,21 | Up |
| A_24_P933305 | A_24_P933305 | 0,29 | 0,53 | 0,85 | 1,21 | Down |
| A_24_P933378 | BF433735 | 0,25 | 0,43 | 0,88 | 1,21 | Down |
| A_24_P934653 | A_24_P934653 | 0,22 | 0,44 | 0,86 | 1,21 | Down |
| A_24_P93624 | ENST00000247833 | 0,11 | -0,16 | 1,20 | 1,21 | Up |
| A_24_P941217 | SGPP2 | 0,00 | 0,15 | 0,90 | 1,21 | Down |
| A_24_P941540 | A_24_P941540 | 0,12 | 0,44 | 0,80 | 1,21 | Down |
| A_24_P942661 | A_24_P942661 | 0,24 | 0,48 | 0,85 | 1,21 | Down |
| A_24_P944478 | A_24_P944478 | 0,17 | 0,32 | 0,90 | 1,21 | Down |
| A_24_P945375 | A_24_P945375 | 0,18 | 0,37 | 0,88 | 1,21 | Down |
| A_24_P96749 | EIF2C1 | 0,11 | 0,39 | 0,82 | 1,21 | Down |
| A_24_P9833 | PRDM11 | 0,25 | 0,46 | 0,86 | 1,21 | Down |
| A_32_P100830 | A_32_P100830 | 0,16 | 0,31 | 0,90 | 1,21 | Down |
| A_32_P104097 | THC2385863 | 0,07 | 0,26 | 0,88 | 1,21 | Down |
| A_32_P105406 | LMBRD2 | 0,50 | 0,75 | 0,84 | 1,21 | Down |
| A_32_P108986 | THC2317817 | 0,20 | 0,39 | 0,88 | 1,21 | Down |
| A_32_P109584 | BC034423 | 0,19 | 0,37 | 0,88 | 1,21 | Down |
| A_32_P113193 | ZNF592 | 0,16 | 0,42 | 0,84 | 1,21 | Down |
| A_32_P119018 | A_32_P119018 | 0,27 | 0,58 | 0,81 | 1,21 | Down |
| A_32_P123788 | LENG4 | 0,16 | 0,36 | 0,87 | 1,21 | Down |
| A_32_P135336 | LOC388242 | 0,27 | 0,47 | 0,87 | 1,21 | Down |
| A_32_P137636 | THC2250668 | 0,15 | 0,36 | 0,86 | 1,21 | Down |
| A_32_P140742 | FLJ30851 | 0,29 | 0,56 | 0,83 | 1,21 | Down |
| A_32_P146485 | THC2287125 | -0,15 | -0,29 | 1,10 | 1,21 | Up |
| A_32_P149432 | EIF4A1 | -1,11 | -0,60 | 0,70 | 1,21 | Down |
| A_32_P150642 | THC2284956 | 0,20 | 0,45 | 0,84 | 1,21 | Down |
| A_32_P162507 | GFRA2 | 0,22 | 0,46 | 0,85 | 1,21 | Down |
| A_32_P164661 | AI302935 | 0,28 | 0,47 | 0,87 | 1,21 | Down |
| A_32_P171984 | A_32_P171984 | 0,17 | 0,56 | 0,76 | 1,21 | Down |
| A_32_P174805 | AI492422 | 0,19 | 0,43 | 0,85 | 1,21 | Down |
| A_32_P207414 | LOC389232 | 0,24 | 0,41 | 0,89 | 1,21 | Down |
| A_32_P210982 | THC2291074 | 0,10 | 0,27 | 0,89 | 1,21 | Down |
| A_32_P223488 | THC2372788 | 0,33 | 0,59 | 0,83 | 1,21 | Down |
| A_32_P226149 | YWHAZ | 0,05 | 0,60 | 0,68 | 1,21 | Down |
| A_32_P229699 | BC019907 | 0,31 | 0,59 | 0,82 | 1,21 | Down |
| A_32_P232383 | THC2442833 | 0,14 | 0,29 | 0,90 | 1,21 | Down |
| A_32_P232437 | A_32_P232437 | 0,06 | 0,22 | 0,89 | 1,21 | Down |
| A_32_P23989 | TSC22D1 | 0,12 | 0,31 | 0,88 | 1,21 | Down |
| A_32_P35486 | THC2317058 | 0,52 | 0,04 | 1,39 | 1,21 | Up |
| A_32_P360518 | CR592225 | 0,25 | 0,47 | 0,86 | 1,21 | Down |
| A_32_P39669 | LOC375010 | -1,40 | -1,82 | 1,34 | 1,21 | Up |
| A_32_P4466 | FLJ32214 | 0,35 | 0,65 | 0,81 | 1,21 | Down |
| A_32_P522060 | BC034299 | 0,08 | 0,24 | 0,89 | 1,21 | Down |
| A_32_P5514 | A_32_P5514 | 0,14 | 0,33 | 0,87 | 1,21 | Down |
| A_32_P5568 | THC2290729 | 0,05 | 0,20 | 0,90 | 1,21 | Down |
| A_32_P58401 | THC2409571 | 0,37 | 0,58 | 0,86 | 1,21 | Down |
| A_32_P709998 | OTOP2 | 0,14 | 0,32 | 0,88 | 1,21 | Down |
| A_32_P718498 | MLLT6 | 0,35 | 0,61 | 0,83 | 1,21 | Down |
| A_32_P73717 | A_32_P73717 | 0,10 | 0,26 | 0,90 | 1,21 | Down |
| A_32_P738283 | AK094642 | 0,03 | 0,19 | 0,89 | 1,21 | Down |
| A_32_P7521 | AA065042 | 0,12 | 0,29 | 0,89 | 1,21 | Down |
| A_32_P79152 | THC2269537 | 0,86 | 1,29 | 0,74 | 1,21 | Down |
| A_32_P86289 | BX091899 | 0,18 | 0,34 | 0,89 | 1,21 | Down |
| A_32_P91633 | AA300289 | -0,08 | -0,27 | 1,14 | 1,21 | Up |
| A_32_P96000 | FLJ16478 | 0,19 | 0,37 | 0,88 | 1,21 | Down |
